# Psychological characteristics in high-risk MSM in China

**DOI:** 10.1186/1471-2458-12-58

**Published:** 2012-01-20

**Authors:** Guanzhi Chen, Yang Li, Beichuan Zhang, Zengzhao Yu, Xiufang Li, Lixin Wang, Ziming Yu

**Affiliations:** 1 Department of Dermatology, Affiliated Hospital of Medical College, Qingdao University, Qingdao, Shandong, China; 2 Department of Dermatology, Prisoner Hospital of Qingdao, Qingdao, Shandong, China; 3 Department of Sex Health Center, Affiliated Hospital of Medical College, Qingdao University, 16 jiangsu Street, Qingdao, Shandong 266000, China; 4 Department of Dermatology, Qingdao Municipal Hospital, Qingdao, Shandong, China; 5 Department of Emergency Medicine, Hainan Medical College-Affiliated Hospital, 31 Longhua Street, Hainan 570102, China

**Keywords:** MSM, HIV infection, Sexual risk behavior, Psychological characteristics, China

## Abstract

**Background:**

Men who have sex with men (MSM) have become a high-risk group of HIV infection in China. To date, little is known regarding the behavioral, social and psychological characteristics in Chinese MSM, which makes the implementation of preventive and therapeutic strategies for this high-risk subpopulation of people extremely difficult.

**Methods:**

A total of 714 questionnaires were retrieved from the database of a Chinese government-sponsored National Key Research Project titled "Risk Analysis and Strategic Prevention of HIV Transmission from MSM to the General Population in China". The respondents were categorized into a high-risk group and a control group. Their behavioral, social and psychological characteristics were comparatively analyzed.

**Results:**

Of the 714 MSM analyzed, 59 (8.26%) had high-risk homosexual behaviors. This sub-group of MSM had a higher in-marriage rate, a higher monthly income, heavier alcohol consumption and more serious problems with sexual abuse in childhood, intentional suicide attempts and mistaken assumption on condom's role in protecting HIV infection, as compared with the control group (*P *< 0.05). In contrast, the two groups did not differ significantly the sexual orientation, level of education, types of profession, drug use, condom use and experience of social stigma and discrimination (*P *> 0.05). A vast majority of the individuals in both behavior categories expressed support of legally protected gay clubs as well as gay marriage legislation in China. There was a strong correlation between high-risk behaviors and sexual abuse in childhood, alcohol drinking, income level and a mistaken belief in perfect HIV protection through the use of condoms.

**Conclusions:**

MSM with and without high-risk homosexual behaviors have different social and psychological characteristics, which should be taken into account when implementing behavioral and therapeutic interventions aimed at preventing HIV/AIDS transmission among MSM as well as from MSM to the general population in China.

## Background

It is estimated that AIDS killed more than 25 million people worldwide from 1981 to 2006 [1]. According to the estimation and statistics of the Joint United Nations Programme on HIV/AIDS (UNAIDS), China remains one of the geographic areas with the lowest overall prevalence of HIV/AIDS in the world. However, there has been an alarming increase in the HIV/AIDS epidemic in recent years in the country [2]. To cope with the increased burden of HIV/AIDS transmission, the Chinese National Center for AIDS/STD Control and Prevention has recommended implementing HIV/AIDS behavioral and therapeutic interventions in China. However, these interventions primarily target drug users and female sex workers through education of HIV-related knowledge and skills, promotion of condom use, increased access to voluntary HIV counseling and testing services, and improved access to treatment for sexually transmitted infections [3]; currently, insufficient attention is being paid to the most-at-risk populations [4].

As one of the most significant changes in the HIV epidemic in recent years in China, MSM have become the group most likely to be infected by HIV. According to a recent estimation, 12.2% of the total HIV infection in 2007 in China occurred among MSM, but by 2009 this proportion robustly rose to 32.5% [5]. To tackle the sharply increased HIV infection rate in this unique subpopulation, "the Chinese government has made addressing HIV prevention among MSM a priority and that is something which UNAIDS welcomes", as stated by Bernhard Schwartlander, UNAIDS Country Coordinator in China [3]. Given the illegal nature of homosexual conduct as well as the prevalent stigmas and discriminations against homosexuality in the general public and even within the MSM community itself in China, it is a significant challenge to reach members of the MSM. As a result, interventions are difficult to be applied to this unique high-risk subpopulation.

Previous studies have shown that sex with multiple gay partners, group sex, commercial gay sex and sex with male strangers are high-risk behaviors for HIV transmission [6]. However, the psychological and social characteristics of the Chinese MSM with these high-risk behaviors as well as factors that may contribute to the development of these behaviors are largely unknown. This study was therefore conducted to assess psychological and social characteristics of MSM with high-risk sexual behaviors across mainland China as well as factors that may contribute to the development of these high-risk behaviors. Our ultimate goals are to develop social and psychological characteristics-based strategies to more effectively identify high-risk Chinese MSM for appropriate interventions as well as to minimize the risk factors underlying the development of high-risk sexual behaviors in Chinese MSM.

## Methods

### Ethical considerations

This study involved MSM, a unique subpopulation of men at high risk of HIV infection. The study protocol, including access and use of the data described below, was critically reviewed and approved by the Institutional Ethics Committee.

### Data access

During the 10th Five-Year Plan (2001-2005) of the People's Republic of China, the Chinese Ministry of Science and Ministry of Health jointly launched and supported a National Key Research Project titled "Risk Analysis and Strategic Prevention of HIV Transmission from MSM to the General Population in China". As part of that project, a questionnaire survey was conducted on MSM in hidden bars in 9 large Chinese cities (i.e. Harbin, Shenyang, Xi'an, Zhengzhou, Shanghai, Nanjing, Wuhan, Chongqing, and Chengdu), using a snowball sampling approach by which potential future subjects were recruited by the existing respondents or subjects from their acquaintances. Based on the retrieved data, the respondents included both urban residents (either by birth or due to employment after college graduation) and rural migrant workers living in the surveyed cities. The execution of the survey strictly followed the ethical principles outlined in the Declaration of Helsinki regarding human clinical research. A written consent signed in person in the snowball sampling process was obtained from each of the subjects. The questionnaire consisted of a total of 214 questions, covering 12 aspects of gay life in China. Data from the returned questionnaires, either totally or partially completed, were stored confidentially in the database of the Project. To investigate the psychological, social, and behavioral characteristics among Chinese MSM, we requested for and were granted conditional access to the Project's database.

### Data retrieval and subject categorization

In the database, there were a total of 2250 returned questionnaires, of which 714 were retrieved for analyses in this study. The exclusion criteria included: 1) "professional" gay sex workers; and 2) failure or refusal to have completely answered the following 3 core questions: 1) Have you ever hunted for male strangers in gay places and had sex with them as well within the last 6 months? 2) Did you keep a sexual relationship with at least 3 male partners at the same time in any time period of last year? 3) Have you ever engaged in commercial sex with homosexual males? Of the 2250 respondents surveyed, 59 answered "yes" to all 3 core questions and were categorized as MSM of high-risk HIV infection, 655 answered "no" to all 3 core questions and were categorized as control MSM, and those who answered "yes" to one or two of the 3 core questions were excluded.

### Statistical analyses

Data were expressed as means ± SD. Differences between groups were analyzed with chi-square test or Mann-Whitney Test. A multiple regression analysis was performed to assess risk factors underlying high-risk behaviors. Differences were considered significant when *P *< 0.05.

## Results

### Demographic characteristics

The average age of the subjects in the high-risk group was 34 (25-42) years, significantly older than that in the control group (25 years, range from 22 to 32) (*P *< 0.01). Other major demographic characteristics of the subjects are presented in Table 1. There was no significant difference in sexual orientation between the two groups (*P *> 0.05; 95% CI: 0.698-2.118). Significantly more subjects in the high-risk behavior group than in the control group were married (*P *< 0.05; 95% CI: 1.197-3.825). Significantly fewer subjects in the high-risk behavior group than in the control group had a monthly wage over RMB2000 (*P *< 0.05; 95% CI: 2.760-8.294). The two groups did not significantly differ in educational backgrounds (*P *> 0.05; 95% CI: 0.835-2.478) and types of professions (*P *> 0.05; 95% CI: 0.204-1.655).

**Table 1:** Basic demographic characteristics of the subjects in the high-risk behavior group (Group A) and the control group (Group B).

	Group A	Group B
Sexual orientation		
Homosexual	38 (64.4%)	393 (60.0%)
Bisexual	21 (35.6%)	264 (40.0%)

Marital status		
Not-in-marriage	40 (67.8%)	536 (81.8%)
In-marriage	19 (32.2%)	119 (18.2%)

Monthly income (RMB)		
≥ 2000	35 (59.3%)	153 (23.4%)
< 2000	24 (40.7%)	503 (76.6%)

Level of education		
High school and below	25 (43.1%)	226 (34.5%)
University and above	33 (56.9%)	429 (65.5%)

Profession type		

Physical labor	4 (7.3%)	75 (11.9%)

Non-physical labor	51 (92.7%)	556 (88.1%)

Not-in-marriage includes single, divorced, and widowed subjects; in-marriage includes married and remarried subjects

### Behavioral characteristics

Presented in Tables 2 and 3 are homosexual behaviors and activities of the MSM analyzed in the present study. Compared with the subjects in the control group, the subjects in the high-risk behavior group: 1) became aware of their homosexuality and had their 1st homosexual intercourse at a significantly younger age (*P *< 0.01); 2) had many more homosexual partners, both in total (*P *< 0.01) and in the last 6 months prior to the questionnaire survey (*P *< 0.01); and 3) had significantly more gay anal sex partners, both in total (*P *< 0.01), and in the last 6 months (*P *< 0.01). A significantly lower (*P *< 0.01) percentage of subjects in the high-risk behavior group were living with a "permanent" male sex partner at the time of the survey than those in the control group. In contrast, a significantly higher (*P *< 0.01) percentage of subjects in the high-risk behavior group had active or passive oral, anal and finger sex as well as abusive-masochistic or sadistic sex with gay partners than those in the control group. The two groups did not differ significantly in drug use (*P *> 0.05), but subjects in the high-risk behavior group drank more alcohol than those in the control group (*P *< 0.01). The percentage of subjects using condoms in their oral and anal sex with gay partners in the last 6 months prior to the survey was similar in both groups (*P *> 0.05). Significantly more members in the high-risk group than in the control group experienced condom failure, including breakage and slippage, in the last 6 months prior to the survey (*P *< 0.01).

**Table 2:** Gay sexual activities in the subjects in the high-risk behavior group (Group A) and the control group (Group B).

	A group	B group	Z value	P value
Age (years) of HS awareness	15 (12-18, n = 59)	17 (14-20, n = 646)	-3.19	0.001
Age (years) of 1^st ^gay intercourse	18 (16-22, n = 59)	20 (18-23, n = 644)	-3.00	0.003
Total homosexual partners	99 (35-200, n = 57)	7 (4-15, n = 650)	-10.70	0.000
Total ASGPs	45 (15.75-100, n = 54)	4 (2-10, n = 573)	-9.27	0.000
HSPs over past 6 months	10 (5-20, n = 59)	1 (1-2, n = 642)	-11.45	0.000
ASGPs over past 6 months	9.5 (3-15.5, n = 50)	1 (1-2, n = 547)	-10.07	0.000

HS: homosexuality; ASGPs: anal sex gay partners; HSPs: homosexual partners

**Table 3:** Behavioral characteristics of the subjects in the high-risk behavior group (group A) and the control group (group B).

	Group A	Group B	χ^2^	P
Permanent male sex partner to live together	43.1% (25/58)	63.3% (395/624)	9.15	0.002

Active or passive oral-anal behaviors	72.9% (43/59)	37.7% (248/657)	27.70	0.000

Active or passive finger-anal behaviors	65.5% (38/58)	41.7% (273/655)	12.31	0.000

Bleeding-associated masochistic behaviors	5.4% (3/56)	2.8% (18/651)	1.20	0.273

Bleeding in sexual intercourse last year	42.1% (24/57)	24.3% (158/651)	8.72	0.003

Condom use in oral sex in last 6 months*	88.5% (46/52)	81.8% (413/505)	1.45	0.225

Condom use in anal sex in last 6 months*	39.2% (20/51)	43.4% (214/492)	0.35	0.557

Condom failure in last 6 months**	25.5% (13/51)	8.3%(42/503)	15.21	0.000

Alcohol use	45.8% (27/59)	26.3% (171/650)	10.17	0.001

Drug use	9.1% (5/59)	4.6% (29/633)	2.19	0.139

Since not all the respondents included in this study answered all the questions in the survey, the sample size varied for different parameters analyzed. * indicated "never or seldom used condoms in the past 6 months prior to the questionnaire survey"; ** indicates to condom breakage and slippage

### Psychological characteristics

Presented in Table 4 are psychological characteristics of the subjects analyzed in the present study. When asked whether they had thought about committing suicide, more respondents in the high-risk behavior group than in the control group answered "yes" (29.8% versus 17.3%, *P *< 0.05). When asked whether they would be willing to attend gay clubs if legally protected and whether they would vote for gay marriage legislation in China, both groups had similar responses (*P *> 0.05); 60-70% of respondents expressed a willingness to go to protected gay clubs and 70-80% respondents were supportive of gay marriage legislation.

**Table 4:** Psychological characteristics of the subjects in the high-risk behavior group (Group A) and the control group (Group B).

	Group A	Group B	χ^2^	P
Strong suicidal thoughts	29.8% (17/59)	17.3%(109/630)	5.47	0.019

Willing to attend gay clubs	69.5%(41/59)	58.0% (379/653)	2.93	0.087

Support of gay marriage	78.0% (46/59)	72.4% (471/651)	0.86	0.353

Belief in unlikelihood of HIV infection	32.2% (19/59)	59.8% (377/630)	16.86	0.000

Belief in condoms against HIV infection	36.8% (21/57)	19.2% (125/650)	9.92	0.002

Belief in condoms against other STDs	96.6% (57/59)	89.2% (580/650)	3.23	0.072

Assumption of no VDs with NASOs	46.4% (26/56)	31.3% (203/649)	5.3	0.020

Offended upon SOD	45.8% (27/59)	52.3% (340/650)	0.93	0.335

Hurt by heterosexual individuals	26.3% (15/57)	11.3% (73/645)	10.74	0.001

Hurt by homosexual gay individuals	32.1% (18/56)	22.8% (147/645)	2.50	0.114

Life-affecting pain from sexual identity	59.6% (31/49)	43.7% (251/532)	3.62	0.057

SAAM before the age of 16 years	63.8% (37/58)	11.1% (73/656)	113.41	0.000

STDs: sexually transmitted diseases; VDs: venereal diseases; NASOs: normal appearance of sexual organs; SOD: sexual orientation disclosure; SAAM: sexual abuse by adult male(s)

Significantly more respondents in the high-risk group than in the control group believed that: 1) male homosexual activities or behaviors were unlikely to increase the risk for HIV infection (*P *< 0.01); and 2) use of condoms could completely (i.e. 100%) prevent HIV infection (*P *< 0.01). A vast majority of the respondents in both groups believed that the use of condoms could effectively prevent other sexually transmitted diseases (*P *> 0.05); more respondents in the high-risk group than in the control group thought that no venereal diseases had be contracted if the appearance of sex organs looked normal (*P *< 0.05).

As shown in Table 4, approximately 50% of the MSM in both groups had experienced insults or forms of discrimination when their homosexual identity was first disclosed, regardless of the HIV infection risk level of their homosexual behaviors and activities (*P *> 0.05). Although the percentage of respondents who had been offended by homosexual individuals was similar for both groups, significantly more respondents in the high-risk behavior group than in the control group had been emotionally hurt by the behavior of heterosexual individuals (*P *< 0.01). Approximately 60% of respondents in the high-risk behavior group and 43.7% of the control group (*P *> 0.05) felt emotional pain associated with their homosexuality and admitted that their life was negatively affected by their sexual orientation. More than 60% of respondents in the high-risk behavior group had experienced sexual abuse (i.e. forced sexual intercourse) by adult males before the age of 16 years. This number was almost 6 times that of the control group (*P *< 0.01).

### Major factors potentially attributable to high-risk behaviors

Multivariate regression analysis showed that 4 major independent factors might be attributable to the occurrence of the three types of high-risk behaviors specified in the three core questions in the questionnaire (Table 5). These include: 1) sexual abuse in childhood (95% CI: 8.22-31.82); 2) heavy alcohol consumption (95% CI: 1.87-7.28); 3) monthly income ≥ RMB2000 (95% CI: 2.15-8.14); and 4) mistaken assumption of 100% protection against HIV infection by the use of condoms (95% CI: 1.47-6.08). Marital status failed to correlate with high-risk behaviors (*P *> 0.05).

**Table 5:** Risk factors for HIV infection-high risk behaviors in MSM in China

Risk factors	Wald	P	95% Confidence	
Sexual abuse in childhood	65.01	0.000	8.22	31.82

Alcohol drinking	14.14	0.000	1.87	7.28

Monthly income ≥ RMB 2,000	17.82	0.000	2.15	8.14

Marital status is in-marriage	0.45	0.504	0.62	2.67

Assumption of 100% HIV protection by condoms	9.09	0.003	1.47	6.08

## Discussion

China has experienced a sharp increase in HIV infection among MSM in recent years. Although behavioral intervention is considered as an effective means of reducing HIV infection among MSM [7], an interventional strategy shown to be effective in one setting, place, or time may not necessarily work in another [8]. Strategies should be tailored for risk-different sub-groups of MSM based on their biomedical, behavioral, social, and psychological characteristics [9,10]. In this study, we attempted to characterize social and psychological differences between MSM with and without high-risk behaviors (i.e. having sex with male strangers, participating in group gay sex, and involvement in commercial homosexual activities) in China.

The analysis of the subjects' general demographic data showed that the high-risk MSM and the control MSM were similar in sexual orientation, level of education and types of profession, but significantly differed in marital status and income (Table 1). The percentage of married MSM was significantly higher in the high-risk behavior group than in the control group, in agreement with the findings from a study involving 6,661MSM in India where married MSM were more likely to have unprotected anal sex [11]. At present, there still exist strong cultural stigmas, prejudice and discrimination against homosexuality in China and many other Asian countries; MSM are subject to great pressures from the family and society. As a result, MSM either try to hide their sexual orientation or choose to marry a woman to shield their high-risk homosexual behaviors. This may explain why more subjects were married in the high-risk behavior group than in the control group. Similar to marital status, there was a significant difference in the monthly income between MSM with and without high-risk behaviors, indicating that relatively higher income may make high-risk behaviors, like commercial sex, more economically possible. No significant difference in condom use was observed between the two groups of MSM in this study (Table 2), in disagreement with a previous observation that lower income was related with unprotected sex behavior [12].

The respondents in the high-risk behavior group had more irregular sexual partners and utilized more diversified modes of gay sexual intercourse (e.g. oral-anal and finger-anal sex) than those in the control group (Table 3). This may be related to the fact that MSM in the high-risk behavior group had more liberal and permissive attitude towards sex. Nevertheless, the exact reason behind this needs to be further elucidated. It is well known that an exchange of blood is one of the risk factors for HIV transmission. The analysis in this study showed a relatively high proportion of MSM members in the high-risk behavior group who experienced bleeding during homosexual sex (Table 3). This might be related to the large number of sexual partners and unusual sexual behaviors (e.g. masochistic or sadistic sex). Bleeding-associated sexual activities may substantially increase the risk of HIV infection and AIDS transmission, but further studies are warranted to assess the degree to which bleeding-associated gay sex may increase the chance of HIV infection in Chinese MSM.

To date, little is known regarding the mental and psychological characteristics of MSM in China. In this study, we demonstrated that approximately half of the Chinese MSM studied had experienced different forms and degrees of embarrassment, stigma or discrimination after disclosure of their homosexual orientation and that 44% to 60% of the respondents felt that their life was painfully affected by their sexual orientation, regardless of high-risk sexual behaviors or not (Table 4). This study also indicated that almost 70% and 80% of the surveyed MSM respectively expressed a willingness to attend gay clubs if legally protected and were supportive of gay marriage legislation in China (Table 4). It has been reported that MSM are more likely to have intentions of suicide, as compared with the general population [13] and that among MSM, suicidal intentions are even stronger among those with high-risk behaviors [14]. In this study, we found that 29.8% of MSM with high-risk behaviors had considered suicide. Although this number was significantly lower in MSM without high-risk behaviors, the rate was still high at 17.3% (Table 4). In China, suicidal thoughts are most likely to result from psychological pressure and failure of being accepted by mainstream society. Although future legal recognition and cultural acceptance of homosexuality may substantially reduce suicide intentions and attempts in MSM in China, these cannot happen overnight. At present, a greater access to psychological counseling services is practical and feasible.

The relationship between the mentality and individual high-risk sex behaviors in MSM has not been well studied [15]. Although a study on Black and Latino MSM has suggested that impulsive decision-making, sensation-seeking, anxiety and depression and internalized homophobia may all contribute uniquely to the behavior of unprotected anal sex [16], more has to be learned about the specific factors that lead to high-risk sexual behaviors in MSM in China in order to minimize the development of these high-risk behaviors and to implement more appropriate and effective behavioral and therapeutic interventions. Physical or sexual abuse in childhood has been identified as a contributing factor in adverse psychological states as well as high-risk sexual behaviors in adulthood [17-19]. Multivariate regression analyses in this study showed that the most important factor attributable to the development of high-risk sexual behaviors in Chinese MSM was sexual abuse from an adult male before the age of 16, followed by heavy alcohol consumption, income level, and the assumption that the use of condoms can completely (100%) prevent HIV infection (Table 5). Our findings are consistent with previous studies which indicated that sexual abuse in childhood [20] (which is more common in countries lacking legal protection of homosexuality [21]), traumatic injury-associated alcohol abuse [22], and alcohol addiction [23] were identified as risk factors for high-risk sexual behaviors. It is therefore highly logical and advisable to minimize the risk of high-risk homosexual behaviors in MSM by implementing a zero tolerance approach to all forms of childhood sexual abuse and publicly discouraging excessive alcohol consumption as previously proposed [24]. Additionally, it is imperative to implement public education campaigns to correct the misunderstanding that the use of condoms can completely protect against HIV/AIDS.

The data analyzed in this study were from a national survey where the respondents were recruited by a snowball sampling approach. Given some limitations (e.g. more subjects recruited into the sample by some upper layer subjects who happen to have more acquaintances of MSM) intrinsically associated with this sampling approach, some unavoidable sampling bias may exist in the study.

## Conclusions

Of the 714 MSM analyzed in the present study, 59 (8.26%) had high-risk homosexual behaviors. A higher in-marriage rate, a higher monthly income, a higher percentage of individuals who had experienced sexual abuse by adult male(s) in childhood, heavier alcohol consumption, a higher percentage of individuals with the mistaken belief of 100% prevention of HIV infection and AIDS transmission by condoms, and a higher percentage of individuals who had committed at least one suicide attempt were observed in the high-risk behavior group, as compared with the control group. In contrast, MSM with and without high-risk homosexual behaviors did not differ significantly in sexual orientation, level of education, types of profession, drug use, condom use and experiences of social stigma and discrimination. Additionally, a vast majority of the individuals in both behavior categories expressed support of legally protected gay clubs as well as gay marriage legislation in China. Taken together, our analyses indicate that MSM with and without high-risk homosexual behaviors have their own unique social and psychological characteristics and that sexual abuse in childhood, alcohol drinking, monthly income and the mistaken assumption of complete protection against HIV infection and AIDS transmission through the use of condoms are major contributing factors for high-risk sexual behaviors in Chinese MSM. All these group-specific social and psychological characteristics as well as contributing factors for high-risk sexual behaviors must be taken into account when developing and implementing behavioral and therapeutic interventions to target MSM, particularly those with high-risk behaviors, in China.

## Competing interests

The authors declare that they have no competing interests.

## Authors' contributions

Guanzhi Chen and Yang Li equally contributed to this work by performing data retrieval and statistical analyses and preparing the initial draft of the manuscript. Beichuan Zhang was the PI of this study and constructed the conceptual framework of the work. Zengzhao Yu participated in the data analyses. Xiufang Li and Lixin Wang offered help for the conception of this paper, and assisted in the completion of writing the paper. Ziming Yu consolidated the intellectual contents of the study, re-constructed and re-wrote the manuscript in a logically clearer and language-wise more proficient manner. All authors have read and approved the final version of the manuscript.

## Pre-publication history

The pre-publication history for this paper can be accessed here:

http://www.biomedcentral.com/1471-2458/12/58/prepub
